# Mendelian randomization: a novel approach for the prediction of adverse drug events and drug repurposing opportunities

**DOI:** 10.1093/ije/dyx207

**Published:** 2017-10-11

**Authors:** Venexia M Walker, George Davey Smith, Neil M Davies, Richard M Martin

**Affiliations:** dyx207-1 MRC Integrative Epidemiology Unit, University of Bristol, Bristol, UK, and; dyx207-2 Bristol Medical School, University of Bristol, Bristol, UK

**Keywords:** Mendelian randomization, pharmacovigilance, drug repurposing, adverse drug events

## Abstract

Identification of unintended drug effects, specifically drug repurposing opportunities and adverse drug events, maximizes the benefit of a drug and protects the health of patients. However, current observational research methods are subject to several biases. These include confounding by indication, reverse causality and missing data. We propose that Mendelian randomization (MR) offers a novel approach for the prediction of unintended drug effects. In particular, we advocate the synthesis of evidence from this method and other approaches, in the spirit of triangulation, to improve causal inferences concerning drug effects. MR addresses some of the limitations associated with the existing methods in this field. Furthermore, it can be applied either before or after approval of the drug, and could therefore prevent the potentially harmful exposure of patients in clinical trials and beyond. The potential of MR as a pharmacovigilance and drug repurposing tool is yet to be realized, and could both help prevent adverse drug events and identify novel indications for existing drugs in the future.

**Key Messages**

- We propose that the prediction of unintended drug effects using MR can overcome some of the limitations associated with existing methods, including confounding by indication, reverse causality and missing data.
- We demonstrate the potential of MR for predicting unintended drug effects using examples from the literature of studies that have assessed recognized unintended drug effects.
- We advocate the synthesis of evidence from MR and other approaches, in the spirit of triangulation, to improve causal inferences concerning drug effects.

## Introduction

Adverse drug events and drug repurposing opportunities are both unintended drug effects. Drug repurposing, defined as the application of known drugs to new indications, offers a time- and cost-effective alternative to traditional drug development.^1^ Adverse drug events, defined as any unwanted reaction to a drug, risk patient safety and increase the burden on health care systems.^2^ The opportunities offered by drug repurposing and the potential harm caused by adverse drug events means the identification of unintended drug effects is necessary to maximize the benefit of a drug and protect health.

Unintended effects of drugs can be discovered throughout the drug development process. However, before approval of a novel drug, its risk-benefit profile cannot be fully known. This is because pre-approval clinical trials are principally for demonstrating the drug’s efficacy for its intended indication. This limits the trial’s ability to assess safety and identify novel indications in a number of ways.^3^ First, the comparatively small number of patients exposed to a drug during a pre-approval clinical trial means that only very common or very large drug effects can be detected. Second, the length of time that patients are exposed to the drug in this setting is relatively short. Third, the recorded data may not include the necessary information to identify previously unknown drug effects or those that are unrelated to the drug’s indication. Finally, the participants of a study may not represent the broad range of patients seen in clinical practice. As a result of this, continued assessment of drugs after approval is necessary in order to fully develop their profile.

After approval of a drug, unintended drug effects can be identified in several ways. Adverse drug events are primarily identified through the use of spontaneous reporting systems, which rely on health care professionals and members of the public to report suspected drug effects.^4–6^ Drug repurposing opportunities are often sought directly by pharmaceutical companies using purpose-built drug repurposing technology platforms, due to their desirable risk-versus-reward trade-off.^7^ Strong signals from these databases and technology platforms are then investigated using data from a range of sources, including: randomized clinical trials (RCTs) either before or after approval of the drug; meta-analyses of such trials; observational studies; and information from basic science.^3^ However these methods, particularly spontaneous reporting systems, suffer from several biases including their inability to determine causality, over-reporting from media coverage, confounding by indication and other usually unobserved confounders. Minimizing these biases is therefore key to determining which of these signals indicate a true unintended drug effect.

## Mendelian Randomization

**Figure 1:**
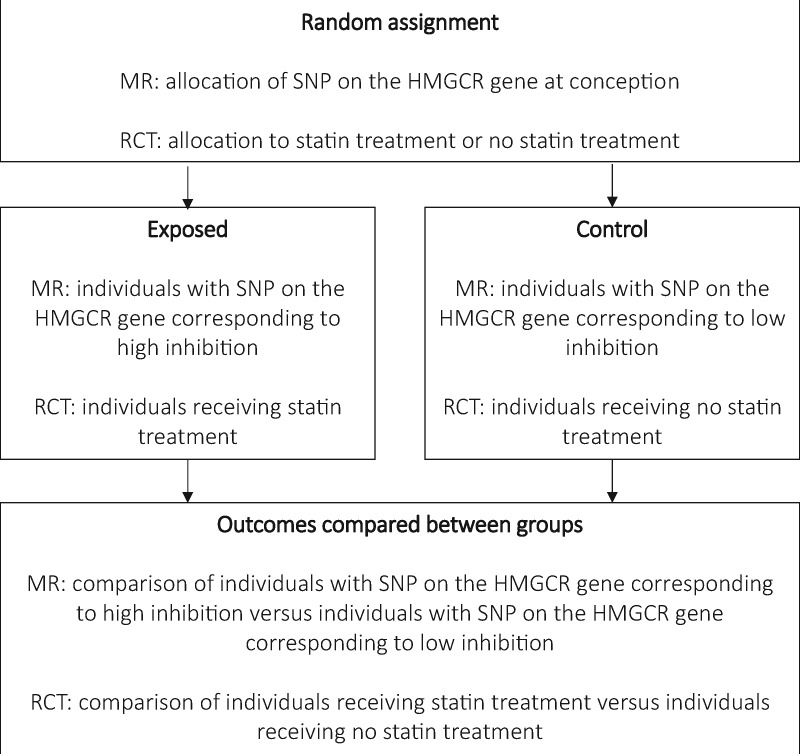
The process by which MR mimics the action of a drug. This diagram shows how MR can be thought of as analogous to an RCT. To predict unintended effects of a drug, the mechanism that the drug alters must be identified so that a suitable proxy for the drug can be identified. Naturally, this mechanism will differ between individuals because of genetic variation. MR therefore uses the random allocation of genetic variants to mimic allocation (or not) to the drug of interest.

We propose that Mendelian randomization (MR) offers a novel approach for the prediction of unintended drug effects, which overcomes some of the limitations associated with existing methods.^8–10^ In particular, we advocate the synthesis of evidence from MR with that from other sources, in the spirit of triangulation, to improve causal inferences of drug effects.^11^ MR assesses the causal effect of an exposure on an outcome by using a genetic variant as a proxy for exposure. For example, MR can interrogate the unintended drug effects associated with statins. Statins inhibit the enzyme 3-hydroxy-3-methylglutaryl-CoA reductase (HMGCR) to lower low-density lipoprotein (LDL) cholesterol and consequently reduce the risk of coronary heart disease (CHD). An MR study would use one or a combination of single nucleotide polymorphisms (SNPs) located in the vicinity of the HMGCR gene as a proxy for exposure to statins. The principle behind this is that randomization occurs naturally at conception, when genetic variants are allocated at random to individuals from their parents. The genetic variants allocated at germ cell formation and conception are part of the germline genome–this is represented in studies by the data collected from routine genotyping. Post-zygotic alterations to the germline can occur in the somatic genome, with such alterations contributing to the development of many cancers and some other diseases. Using germline genetic variants for the prediction of unintended drug effects has a number of strengths and limitations associated with it, which are discussed in detail later.^12^ MR can therefore be thought of as analogous to an RCT that uses genetic variation as the method of randomization, as demonstrated in Figure 1. The key distinction is that MR can be done using routine genotyping data, without the exposure of patients to the drug.

Potential unintended drug effects include: drug substance specific effects; mechanism effects; and biomarker effects. These effects are best presented in terms of the statin example discussed previously. Drug substance specific effects are effects that relate only to the particular drug compound received–this means different compounds within a class of drugs produce different effects. For example, it has been suggested that there is an increased risk of fatal rhabdomyolysis associated with cerivastatin compared with other statins, and this has led to it being withdrawn from the market.^13–17^ Mechanism effects are effects resulting from changes to a specific enzyme or biological pathway but not changes resulting from the biomarker. In terms of statins, this would be changes resulting from the inhibition of HMGCR and not those resulting from changes in LDL cholesterol.^18–20^ For example, multiple statins may have lipid-independent effects resulting from HMGCR inhibition. These effects include improvement of endothelial function, though there is limited direct evidence for this in humans at present.^20–22^ Finally, biomarker effects are the effects that result from changes in the biomarker, i.e. changes in LDL cholesterol level, which occur regardless of the mechanism used to induce that change. For example, reduced LDL cholesterol appears to increase the risk of type 2 diabetes independent of the mechanism of LDL reduction.^23–26^ This has been demonstrated by Ference *et al.*, who found that for three mechanisms–HMGCR, proprotein convertase subtilisin/kexin type 9 (PCSK9) and low-density lipoprotein receptor (LDLR)–‘each set of gene-specific variants … had a very similar effect as the other sets on the risk of diabetes per unit decrease in the LDL cholesterol level’.^27^ Understanding the difference between effects is key to understanding what is possible with MR in this context.

## Examples

The potential of MR for predicting unintended drug effects in the future is highlighted by studies that have assessed recognized unintended drug effects. We will consider two examples: one for the prediction of adverse drug events and the other for the prediction of drug repurposing opportunities. In the case of the former, consider once more the example of statins prescribed for the prevention of CHD. Statins increase the risk of new-onset type 2 diabetes—a risk that is recognized by both the Medicines and Health Care Products Regulatory Agency (MHRA) in the UK and the Food and Drug Administration (FDA) in the USA.^28^^,^^29^ This risk was originally assessed using evidence from a meta-analysis of randomized statin trials.^25^ Since the recognition of this adverse drug event, Swerdlow *et al.* conducted an MR study to assess whether the increase in new-onset type 2 diabetes risk is a result of the inhibition of HMGCR, i.e. the enzyme targeted by statins. To do this, they used the SNP rs17238484 as a proxy because it is located on the HMGCR gene and has been associated with lower LDL cholesterol in a large genome-wide study of lipids.^24^^,^^30^ Swerdlow *et al.* found ‘each additional rs17238484-G allele was associated with a mean 0·06 mmol/l [95% confidence interval (CI) 0.05–0.07] lower LDL cholesterol and higher body weight (0·30 kg, 0.18–0.43), waist circumference (0.32 cm, 0.16–0.47), plasma insulin concentration (1.62%, 0.53–2.72) and plasma glucose concentration (0.23%, 0.02–0.44)’.^23^ This led them to conclude that inhibition of HMGCR ‘at least partially’ explains the increased risk of type 2 diabetes. In principle, MR could potentially have provided evidence of this effect before licensing and before the exposure of large numbers of patients. In this case, MR could also have predicted the balance of benefits and risks of statin treatment in terms of CHD reduction and type 2 diabetes increase (which generally show an overall markedly predictable effect).^16^

The second example focuses on the potential of MR for predicting drug repurposing opportunities. It is thought to take around 10 years from the point where a drug is first tested in humans to the point where it is a licensed treatment.^7^^,^^31^^,^^32^ This means we are yet to see the full benefit of the results from large-scale genome-wide association studies (GWAS) being made available for drug development. Nonetheless, there are several recent examples that highlight the future possibilities. For example, consider serum calcium and the risk of migraine. A study by Yin *et al.* recently investigated this relationship by implementing three methods, including an MR analysis using a genetic score that explained 1.25% of variation in serum calcium levels. Based on this score they found ‘an elevation of serum calcium levels by a hypothetical 1 mg/dl … . was associated with an increase in risk of migraine [odds ratio (OR) 1.80, 95% CI 1.31–2.46, *P* = 2.4 x 10^−4^]’, which was supported by their other two methods.^33^ The paper then went on to highlight several therapeutic options that may be possible based on this evidence. These included the use of the drug Cinacalcet, which is already approved by the FDA, to antagonize the calcium-sensing receptor (CaSR). This drug was suggested based on the variant rs1801725, which is in the CASR gene and associated with both serum calcium levels and increased migraine susceptibility. The authors advised caution due to hypocalcaemia risk, but indicated that Cinacalcet may be a drug repurposing opportunity worth investigating in ‘specific instances’. Another potential therapeutic option arising from this study related to the use of calcium channel blockers (CCBs). Although existing evidence is mixed for the use of these drugs for migraine, the authors suggested that the vasodilatory effects of CCBs accompanied by direct manipulation of Ca^2+^ levels could be beneficial based on their findings.

Further opportunities to predict unintended drug effects are detailed in Table 1. Recent work by Finan *et al.*, to estimate the druggable genome, termed 144 licensed drug targets as having a ‘discordant disease association and target indication considered to imply a potential repurposing opportunity’. A further 27 licensed drug targets were termed as having a ‘disease association corresponding to a mechanism-based adverse effect’.^34^ This work, which used an MR approach for the study of drug effects across the whole genome, along with the selected examples presented in Table 1, illustrate the immense opportunity provided by this method.

**Table 1:** Opportunities to predict unintended drug effects using MR with potential genetic variants identified from the GTEx eQTL Catalog^74^ using MR-Base^45^

Drug	Potential proxy genetic variant(s)	Mechanism	Biomarker	Target disease
Aldehyde dehydrogenase inhibitors	rs201649047	Acetaldehyde dehydrogenase	Acetaldehyde	Alcohol dependence
	rs11066055			
	rs592967			
	rs11608345			
	rs111900779			
	rs7963329			
	rs57186456			
	rs201574057			
	rs847892			
	rs200037659			
	rs11066018			
Angiotensin-converting enzyme inhibitors	rs4311	Angiotensin-converting enzyme	Blood pressure	Hypertension
	rs6504163			
	rs4277405			
	rs4330			
Carbonic anhydrase II inhibitors	rs11329721	Carbonic anhydrase II	Intraocular pressure	Open-angle glaucoma
	rs10090196			
	rs3839863			
	rs13282987			
	rs62512073			
	rs79597773			
Cholesteryl ester transfer protein inhibitors	rs821840	Cholesteryl ester transfer protein	Low-density lipoprotein cholesterol	Coronary heart disease
	rs11508026			
	rs201940645			
Ezetimibe	rs411279633	Niemann-pick C1-like 1	Low-density lipoprotein cholesterol	Coronary heart disease
	rs199683176			
	rs217402			
	rs11972520			
	rs745833			
Fatty acid amide hydrolase inhibitors	rs7520850	Fatty acid amide hydrolase	Anandamide	Inflammatory chronic pain
	rs6429600			
	rs2145409			
	rs7555240			
	rs2145409			
	rs2145409			
	rs6429600			
	rs56083025			
	rs35361357			
	rs56083025			
	rs6429600			
	rs7555240			
	rs4660346			
	rs2145409			
	rs56083025			
	rs11804189			
	rs12217016			
	rs201127808			
Gonadotrophin-releasing hormone antagonists	rs28526365	Gonadotropin-releasing hormone receptors	Luteinising hormone	Prostate cancer
	rs12651577			
	rs145250522			
	rs17634475			
	rs12651577			
	rs141552662			
	rs147425774			
	rs398107462			
	rs145250522			
	rs11283415			
	rs199604647			
	rs147425774			
	rs1484186			
	rs71219068			
	rs13124793			
	rs11282189			
Proprotein convertase subtilisin/ kexin type 9 inhibitors	rs2495503	Proprotein convertase subtilisin/kexin type 9	Low-density lipoprotein cholesterol	Coronary heart disease
	rs34232196			
	rs479910			
Selenium	rs673752	Dimethylglycine dehydrogenase	Plasma selenium	Prostate cancer
	rs28326			
	rs7714738			
	rs7356546			
	rs146701923			
	rs72764983			
	rs248381			
	rs485851			
	rs6453427			
	rs684277			
	rs1717567			
	rs1274984			
	rs7719892			
Statins	rs17244897	3-hydroxy-3-methylglutaryl-coenzyme A reductase	Low-density lipoprotein cholesterol	Coronary heart disease

Several of these drugs have already been the subject of MR studies, including ezetimibe and statins.^23^^,^^27^^,^^61^ However, these drugs could still benefit from further research, particularly combining MR with a ‘phenotype screen’ (MR-PheWAS) in order to generate hypotheses.^41^

## Existing Genetic Methods

The use of genetics for pharmacovigilance and drug repurposing has previously been discussed; however, the potential of MR for this purpose is yet to be fully realized.^35^^,^^36^ Until now the discussion has focused on the use of genome- and phenome-wide association studies (GWAS and PheWAS, respectively).^37–40^ GWAS search for the genetic variants associated with a given phenotype, and PheWAS search for phenotypes associated with a given genetic variant. In these studies, the genetic variant will be a proxy for the exposure and the phenotype will be an unintended drug effect. MR extends the use of genetics for pharmacovigilance and drug repurposing, as it can either be used on a single outcome, or combined with a ‘phenotype screen’ for the prediction of effects of drugs on a wide range of outcomes. The concept of MR with phenotype screening was first introduced by Millard *et al.*, who proposed MR-PheWAS. MR-PheWAS uses ‘automated screening with genotypic instruments to screen for causal associations amongst any number of phenotypic outcomes’.^41^ This approach is hypothesis-free and could therefore be of great use for generating hypotheses concerning potential unintended drug effects, particularly before approval of a drug.

Limited phenotypic screening with MR has previously been demonstrated in the literature by the Interleukin 1 Genetics Consortium in their investigation of the long-term effects of interleukin 1 (IL-1) inhibition.^42^ This study used a GWAS in order to inform the construction of a genetic score. The score combined the information for two SNPs, rs6743376 and rs11687782, which were upstream of the 1L1RN gene and had been shown in the GWAS to be independently associated with circulating IL-1 receptor antagonist concentration.^43^ The study concluded that ‘human genetic data suggest that long-term dual IL-1α/β inhibition could increase cardiovascular risk and, conversely, reduce the risk of development of rheumatoid arthritis’.^42^ Note that the results of this study do not necessarily extend to inhibition of IL-1β alone–an RCT of which recently found a reduced risk of cardiovascular events.^44^ Since this study, the development of databases of harmonized summary GWAS results, such as MR-Base [http://www.mrbase.org/], has made the implementation of MR in this way much simpler.^45^ The use of MR therefore has immense potential for the prediction of adverse drug events and drug repurposing opportunities, with or without a priori hypotheses. Furthermore, MR and the related genetic methods can be used with non-genetic approaches in order to better explore the relationship between the genome and phenome.^46^ Bush *et al.* discuss how genetic data can be linked with data from electronic health records and epidemiological studies in order to better characterize ‘the impact of one or more genetic variants on the phenome’ in the PheWAS setting.^47^ An MR-PheWAS that implemented such an approach could be a particularly powerful tool for the prediction of unintended drug effects.

## Strengths and Limitations

MR has a number of strengths and limitations associated with its use, which are summarized in Table 2. In the following sections, we will highlight some of the strengths that make MR particularly suited to the prediction of unintended drug effects, as well as the limitations that it may be susceptible to in this context.

**Table 2:** Strengths and limitations associated with MR

**Strengths**	Addresses confounding by indication More robust to non-genetic confounding More robust to reverse causation Can be used either before or after approval of a drug Able to predict combined effects of drugs Aids the distinction of mechanism and biomarker effects Addresses missing data Limits associative selection bias^a^ Minimizes regression dilution bias^a^
**Limitations**	Rare effects may not be detected Choice of genetic variant can lead to missed effects or conflicting results^a^^,^^b^ Horizontal pleiotropy Estimates are of lifelong exposure Lack of genetic variants concerning disease progression Unintended drug effects must have large genetic association studies available Genomic confounding Weak instrument bias^a^ Linkage disequilibrium (non-independence of genetic variants)^a^ Combining genetic variants within a model can confound results^a^

^a^These strengths and limitations are not discussed in detail here, but further information can be found in the referenced literature. ^9^^,^^12^^,^^50^^,^^73^

^b^We discuss in detail how the choice of genetic variant can lead to missed effects; however, it may also lead to conflicting results. This can happen if the chosen genetic variant alters the relationship between the exposure and the biomarker or affects multiple biomarkers related to a single disease.

### Strengths

#### Addresses confounding by indication

Confounding by indication occurs in observational studies when the factors predisposing a patient to receive treatment are also the factors related to an increased risk of experiencing an outcome.^48^ This can induce an artificial association between the drug exposure and an observed outcome. MR minimizes confounding by indication because the genetic variant used to proxy drug exposure is unlikely to be affected by the indications for such drug exposure. Let us continue with the example of statins prescribed for the prevention of CHD: existing cardiovascular disease is a major indication for taking statins. At the same time, patients with cardiovascular disease are also at increased risk of death. This can induce an observational association between statin use and increased risk of cardiovascular death. But this association is not caused by statins, it is due to the indication: risk of cardiovascular disease.^49^ MR reduces confounding by indication as the SNP located on the HMGCR gene, used to proxy exposure to statins, is a germline variant and so is unlikely to be a result of the indication, i.e. cardiovascular disease.

#### More robust to non-genetic confounding and reverse causation

MR uses germline genetic variants that are less likely to be confounded by environmental, lifestyle or disease-related factors operating later in life.^50–52^ Consequently, if a genetic variant is associated with an outcome only through its association with a drug effect, it is likely to be because the genetic variant causes the outcome.^53^ Thus, MR should provide robust evidence about the causal effects of intervening on specific biological pathways. This is particularly important when considering physiological factors that change over the life course, such as LDL cholesterol and estrogen levels, because the association of such factors with the outcome is likely to be heavily confounded by environment and lifestyle factors, as well as potentially being subject to reverse causation. For example, epidemiological studies have previously suggested that hormone replacement therapy (HRT) could be protective against CHD. Results from these studies are summarized in a meta-analysis by Stampfer *et al.*, which found the relative risk to be 0.56 (95% CI 0.50–0.61).^54^ However, these results are contrary to a number of clinical trials.^55^ Lawlor *et al.* suggest that a possible explanation for this contradiction is the effect of early life socioeconomic position. They found ‘adverse socioeconomic factors from across the life course were associated with use of HRT’, in a study using data from the British Women’s Heart and Health Study.^56^ An MR study, which should not be subject to bias caused by socioeconomic position at any point in the life course, has since been conducted using data from young women in Hong Kong and older women in the Guangzhou Biobank Cohort Study. Unlike the observational studies, the MR analysis was in line with the results of the clinical trials and concluded that ‘genetically higher 17 b-estradiol was not associated with any cardiovascular disease-related risk factor or with Framingham score (0.01, 95% confidence interval = −1.34 to 1.31).’^57^ This MR analysis therefore confirms that HRT is unlikely to be a suitable drug-repurposing candidate for CHD, without concerns about bias due to socioeconomic position.

#### Can be used either before or after approval of a drug

MR does not require the exposure of patients to the drug–this means it can be implemented at any point during the drug development process and beyond. This can: increase the efficiency of drug development by identifying unsuitable targets; allow pre-specification of likely adverse outcomes in trials; and reduce the possibility of exposing patients to unnecessary risks and harm. For example, consider the potential use of selenium dietary supplements for the prevention of prostate cancer. The Selenium and Vitamin E Cancer Prevention Trial (SELECT) found that selenium did not lower prostate cancer risk but did increase the risk of type 2 diabetes. An MR study, conducted after the trial, found that genetically elevated selenium was not associated with prostate cancer risk and was positively associated with type 2 diabetes risk (Martin RM, personal communication).^58^ Implementation of MR before the trial could therefore have been an informative step in the assessment of selenium as a possible chemoprevention target.

#### Able to predict combined effects of drugs

Many medicines are only licensed for use when other treatments are either being used concurrently or have been previously used and failed. This makes the assessment of the ‘additive’ effect of drugs increasingly important. For example, the results of the Further Cardiovascular Outcomes Research with PCSK9 Inhibition in Subjects with Elevated Risk (FOURIER) trial, for the PCSK9 inhibitor evolocumab to reduce LDL cholesterol, have recently been published.^59^ This trial was conducted against a background of taking statins. Before this and other PCSK9 inhibitor trials, there had been concerns that PCSK9 inhibitors may have similar effects in terms of type 2 diabetes risk as statins. This led to several MR studies being conducted, including an analysis by Ference *et al.* that examined this risk using variation in PCSK9, HMGCR or both.^27^ The MR study found PCSK9 variants to have a similar effect as HMGCR variants on the risk of cardiovascular events (OR 0.81, 0.74–0.89 vs OR 0.81, 0.72–0.90) and the risk of type 2 diabetes (OR 1.11, 1.04–1.19 vs 1.13, 1.06–1.20) for each 10 mg per dl decrease in LDL cholesterol level. The trial found that the lipid-lowering effect of the PCSK9 inhibitor evolocumab was in line with statins and ‘the rates of adjudicated cases of new-onset diabetes did not differ significantly between the two groups (hazard ratio, 1.05; 95% CI, 0.94 to 1.17)’.^59^ In addition, both the trial and the MR analysis were in agreement that the lipid-lowering effect of statins and PCSK9 inhibitors is additive. Given these overlapping results, further trial data are required to assess the risk of type 2 diabetes associated with the use of PCSK9 inhibitors. However, the consistency of the MR results and the trial clearly demonstrates the value of MR analyses and, in particular, the ability to consider the combined effects of drugs before patient exposure. 

#### Aids the distinction of mechanism and biomarker effects

**Figure 2:**
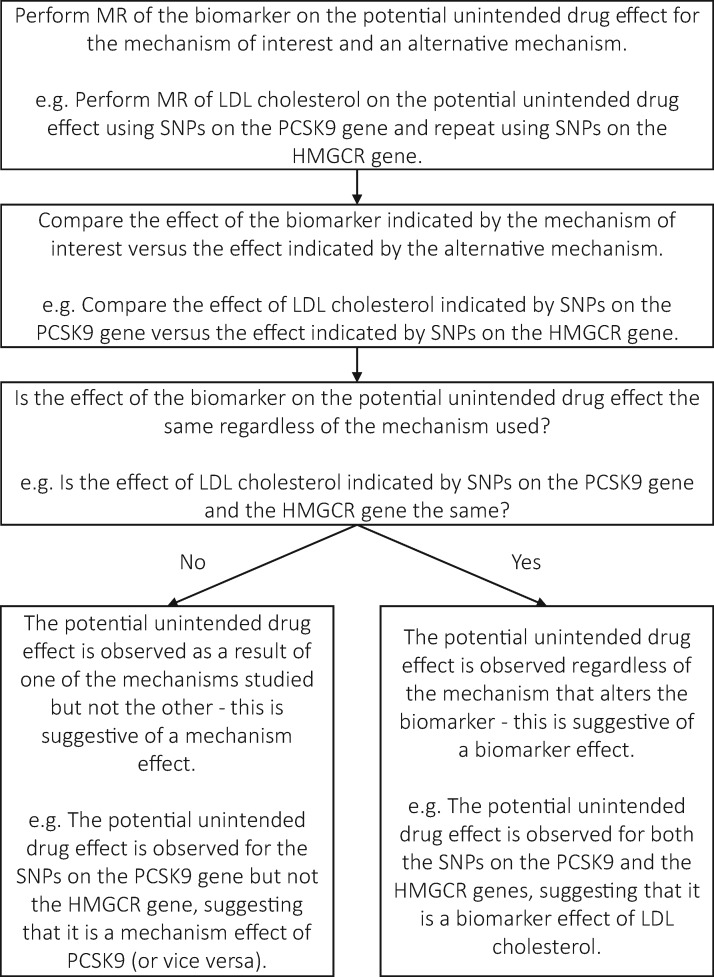
The process by which MR can be used to distinguish mechanism and biomarker effects of drugs. This diagram shows that if a potential unintended drug effect is indicated by the SNPs on multiple genes, then it is suggestive of a biomarker effect. This is because the effect occurs regardless of the mechanism used to induce the change. If this is not the case, the unintended drug effect is suggestive of a mechanism effect relating to the SNPs that indicated it. This is because the effect is specific to just one mechanism that induces a change in the biomarker, and not all possible mechanisms.

MR is able to distinguish mechanism and biomarker effects by enabling a formal statistical comparison of the effect of a biomarker influenced by different drug-related mechanisms to be made.^20^^,^^60^^,^^61^ This process is illustrated in Figure 2. Unfortunately, genetic variants that proxy specific drug substances are rare, and so it is difficult to distinguish drug substance specific effects from the other effect types. MR suggests that an effect is mechanistic if genetic variants for a biomarker mediated by one mechanism affect a downstream phenotype, but variants that are mediated via an alternative mechanism do not. If all variants for a biomarker affect the downstream phenotype regardless of the mechanism, then this suggests a biomarker effect. For example, Ference *et al.* suggested that the cause of increased type 2 diabetes risk may be related to an LDL receptor-mediated pathway, i.e. may be a biomarker effect, based on the study described earlier. This was due to the similarity of the variant effects for HMGCR, the mechanism of statins, and those for PCSK9, the mechanism of PCSK9 inhibitors–along with specific assessment of potential shared pathways.^27^ Other genetic studies have found similar results.^26^^,^^27^^,^^62^

#### Addresses missing data

The use of genetic variants to proxy an exposure in MR can address missing or incomplete exposure, outcome or confounder data. GWAS are increasingly publishing the associations between all genetic variants and their outcome. This means that the associations between a genetic variant and an outcome can be looked up in databases of GWAS results, such as MR Base [http://www.mrbase.org/], and the need for new analysis of individual-level data is removed.^45^ Provided there are robust genetic variants for the drug exposure of interest (see Table 1 for examples) and there are large genetic association studies of the outcome, MR should not be limited by missing or incomplete data.

### Limitations

#### Genomic confounding

MR can be subject to genomic confounding, which occurs when the causality of a genetic variant is misinterpreted. An example of this is population stratification. Genetic variants occur at different frequencies in different populations. This means if, for instance, different ethnicities have different rates of outcomes, differences due to ethnicity could be incorrectly ascribed to the risk factor of interest.

#### Rare effects may not be detected

Single-sample MR studies, where the instrument-therapeutic target associations and the instrument-unintended drug effect associations are recorded in the same dataset, are not suited to detecting rare unintended drug effects. This is due to the power and data availability issues associated with such effects. For example, it has been suggested that rhabdomyolysis may be a mechanism effect of statins that is more pronounced for cerivastatin, rather than a drug-specific effect of cerivastatin. The global incidence of rhabdomyolysis is unknown but it is thought to be rare, with an estimated 26 000 cases per year occurring in the USA according to the 1995 National Hospital Discharge Survey.^64^^,^^65^ This means that single-sample MR studies are unlikely to have sufficient power to detect rhabdomyolysis as a mechanism effect of statins.^66–69^ Two-sample MR studies, which use results from large GWAS for the instrument-therapeutic target associations and case-control GWAS for the instrument-unintended drug effect associations, may be the best approach to overcome this limitation. Note that rhabdomyolysis is a suspected rare unintended drug effect. Investigation of previously unknown rare unintended drug effects would require a hypothesis-free approach. Although this is theoretically possible, it will be hard to achieve with the currently available resources. Curation of a database of GWAS for classical rare unintended drugs effects that drugs could be tested against, in an MR framework, would make such investigations more feasible.

#### Choice of genetic variant can lead to missed effects

Unintended drug effects may be missed if you chose a genetic variant to proxy exposure downstream of the effect you are interested in. For example, if you chose a genetic variant at the biomarker level (i.e. related to LDL cholesterol level) to investigate statins, then the mechanism effects (i.e. the lipid-independent effects such as improved endothelial function) may be missed. In addition to this, genetic association studies often investigate only common genetic variants or combine the effect of rare genetic variants. This results in a situation where individual genetic variants may explain very little of the observed variation. Careful consideration must therefore be given to the choice of genetic variant when conducting an MR study.

#### Horizontal pleiotropy

Horizontal pleiotropy, where a genetic variant influences multiple phenotypes through distinct pathways, is a particular concern for MR analyses.^10^ This can occur if you choose a genetic variant that relates to multiple biomarkers on different pathways, which could affect the outcome of interest. This is because the MR estimate will be biased by the effect of the variant on biomarkers other than the biomarker of interest. Some methods are potentially more robust to certain types of pleiotropy, such as the weighted median and MR-Egger regression methods: these methods are discussed in detail elsewhere.^70^^,^^71^

#### Estimates are of lifelong exposure

MR estimates indicate lifelong perturbations in an exposure. Therefore, careful consideration of the exposure and its timing must be made to avoid misinterpretation of results.^12^^,^^72^ For example, some exposures are cumulative whereby repeated exposure, over a sustained period, results in the outcome. MR analyses of such exposures are likely to overestimate the effect observed in other study designs, including RCTs, as these designs consider much shorter periods of exposure with lower compliance. A further example is time-dependent exposures. MR analyses of this type of exposure can provide misleading evidence about the effect of manipulating an exposure after the critical period. This is because the MR estimate will, by definition, include any critical periods in its assessment of lifelong exposure.

#### Lack of genetic variants concerning disease progression

A large proportion of the genetic variants that have been identified to date are concerned with the incidence of disease. In order to predict unintended drug effects that relate to the treatment of that disease, genetic variants relating to progression will need to be identified. Paternoster *et al.* recently commented that ‘Only a small proportion of GWAS studies [∼8% of associations curated in the GWAS Catalog (*P* < 1 x 10^−5^)] have attempted to identify variants associated with disease progression or severity and those that have are mostly small (90% have *n* < 5000)’.^73^ This limits the treatments we can study at present using MR; however, this can be rectified with increased focus on large GWAS concerning disease progression in the future.

## Conclusion

MR offers a novel and appealing approach for the prediction of unintended drug effects, which can address some of the limitations associated with existing methods in this field. In addition, MR can provide additional benefits, such as use before approval of a drug, and be used in triangulation with evidence from other methods to improve causal inferences concerning unintended drug effects. The potential of MR as a pharmacovigilance and drug repurposing tool is yet to be realized. Future use of this method in the development of drug profiles could both help prevent adverse drug events and identify novel indications for existing drugs.

## Funding

This work was supported by the Perros Trust and the MRC Integrative Epidemiology Unit. The MRC Integrative Epidemiology Unit is supported by the Medical Research Council and the University of Bristol [grant number MC_UU_12013/1, MC_UU_12013/9].

**Conflict of interest:** V.M.W. is the recipient of an Elizabeth Blackwell Institute Proximity to Discovery Award, which involves conducting a research project with GlaxoSmithKline. The project is funded by the Elizabeth Blackwell Institute, University of Bristol and the Medical Research Council.
